# A Case Report of Right Phrenic Nerve Palsy Following Pulsed Field Ablation in a Patient with Persistent Atrial Fibrillation

**DOI:** 10.19102/icrm.2026.17082

**Published:** 2026-08-15

**Authors:** Hasan Munshi, Abdullah Ahmad, Abdel Dajani, Own Khraisat, Amer Hammad, Satish Tiyyagura

**Affiliations:** 1 Department of Internal Medicine, St Joseph University Medical Center, Paterson, NJ, USA; 2 Department of Cardiology, St Joseph University Medical Center, Paterson, NJ, USA; 3 Department of Internal Medicine, Englewood Hospital and Medical Center, Englewood, NJ, USA

**Keywords:** Phrenic nerve injury, posterior wall isolation, pulmonary vein isolation, pulsed field ablation

## Abstract

Pulsed field ablation (PFA) is a nonthermal ablation modality that achieves myocardial ablation through irreversible electroporation and has been associated with a reduced risk of collateral tissue injury. Phrenic nerve injury is uncommon with PFA, and persistent injury has rarely been described. A 61-year-old woman with symptomatic persistent atrial fibrillation refractory to anti-arrhythmic drug therapy underwent catheter ablation using the PulseSelect™ PFA system (Medtronic, Minneapolis, MN, USA). Circumferential PFA applications were delivered to all four pulmonary veins, followed by additional applications to the posterior left atrial wall to achieve posterior wall isolation. A total of 61 pulsed field energy applications were delivered, and the entrance and exit blocks of the pulmonary veins and posterior wall were confirmed. The procedure was otherwise uncomplicated. Several hours post-procedure, the patient developed new-onset dyspnea. Chest radiography demonstrated elevation of the right hemidiaphragm consistent with right phrenic nerve palsy. Conservative management was initiated, with gradual symptomatic improvement. However, follow-up imaging at 3 months demonstrated persistent right hemidiaphragmatic elevation. This case demonstrates that phrenic nerve palsy may occur following pulmonary vein and posterior wall isolation using PFA. Continued awareness of this potential complication and careful post-procedural follow-up are warranted as clinical experience with PFA continues to expand.

## Introduction

Pulsed field ablation (PFA) is an emerging nonthermal ablation modality designed to achieve myocardial-specific tissue ablation through irreversible electroporation, minimizing damage to adjacent structures.^1^ Large clinical trials have demonstrated its noninferiority to thermal ablation techniques with significantly fewer adverse events.^2,3^ However, phrenic nerve injury, though rare, remains a potential risk.^4^ Studies have shown an association with PFA energy and the location of the phrenic nerve in relation to the catheter that may influence the risk of injury to the nerve.^5^ We report a rare case of right phrenic nerve (RPN) palsy with persistent hemidiaphragmatic elevation at 3-month imaging follow-up, following pulmonary vein isolation (PVI) and posterior wall isolation using a PulseSelect™ catheter (Medtronic, Minneapolis, MN, USA) for PFA in a patient with paroxysmal atrial fibrillation.

## Case presentation

A 61-year-old woman with a history of persistent atrial fibrillation, which was first diagnosed in 2020; hypertension; hypothyroidism; and prior coronavirus disease 2019 infection in 2019 presented with recurrent symptomatic episodes of atrial fibrillation despite anti-arrhythmic therapy. She refused amiodarone due to concerns over side effects. She had previously tried 100 mg of flecainide twice daily, which was reduced to 50 mg twice daily due to dizziness. She was initially anticoagulated with apixaban and later switched to dabigatran for affordability reasons.

**Figure 1:**
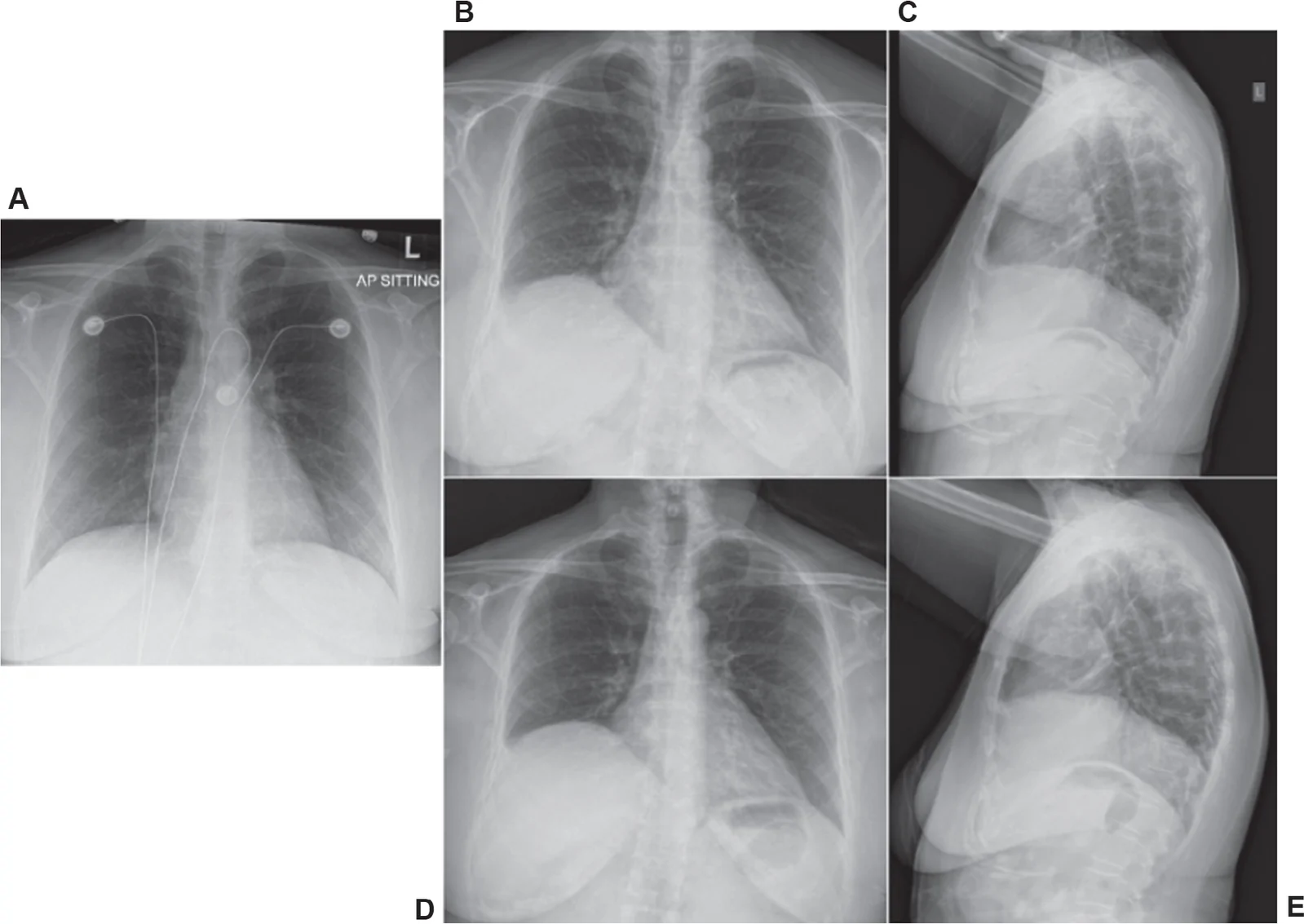
Chest X-ray images. **A:** Normal antero-posterior chest radiograph pre-ablation. **B:** Post-procedural antero-posterior chest radiograph showing right hemidiaphragm elevation consistent with diaphragm paralysis. **C:** Post-procedural lateral chest radiograph showing right hemidiaphragm elevation consistent with diaphragm paralysis. **D:** Post-procedural 3-month follow-up antero-posterior chest radiograph showing right hemidiaphragm elevation consistent with diaphragm paralysis. **E:** Post-procedural 3-month follow-up lateral chest radiograph showing right hemidiaphragm elevation consistent with diaphragm paralysis.

Her pre-procedural workup included a normal echocardiogram. A baseline electrocardiogram (ECG) revealed atrial fibrillation. Her pre-procedural chest radiograph was normal **(Figure 1)**. She was scheduled for catheter ablation using PFA technology.

**Figure 2:**
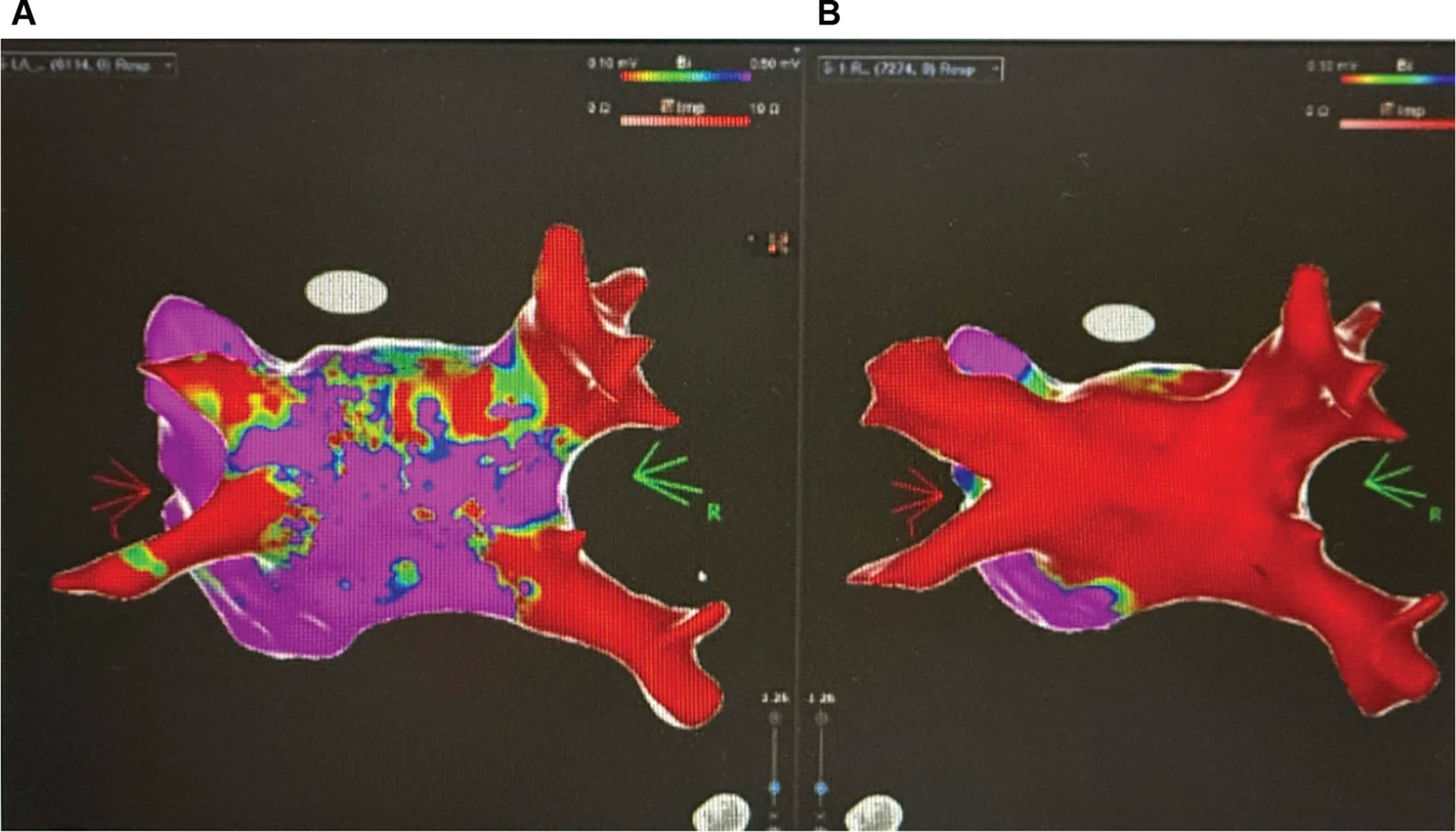
Voltage mapping. **A:** Pre-ablation voltage mapping showing heterogeneous atrial substrate with preserved and low-voltage areas. **B:** Post-ablation voltage mapping demonstrates extensive low-voltage consistent with successful lesion ablation.

**Figure 3:**
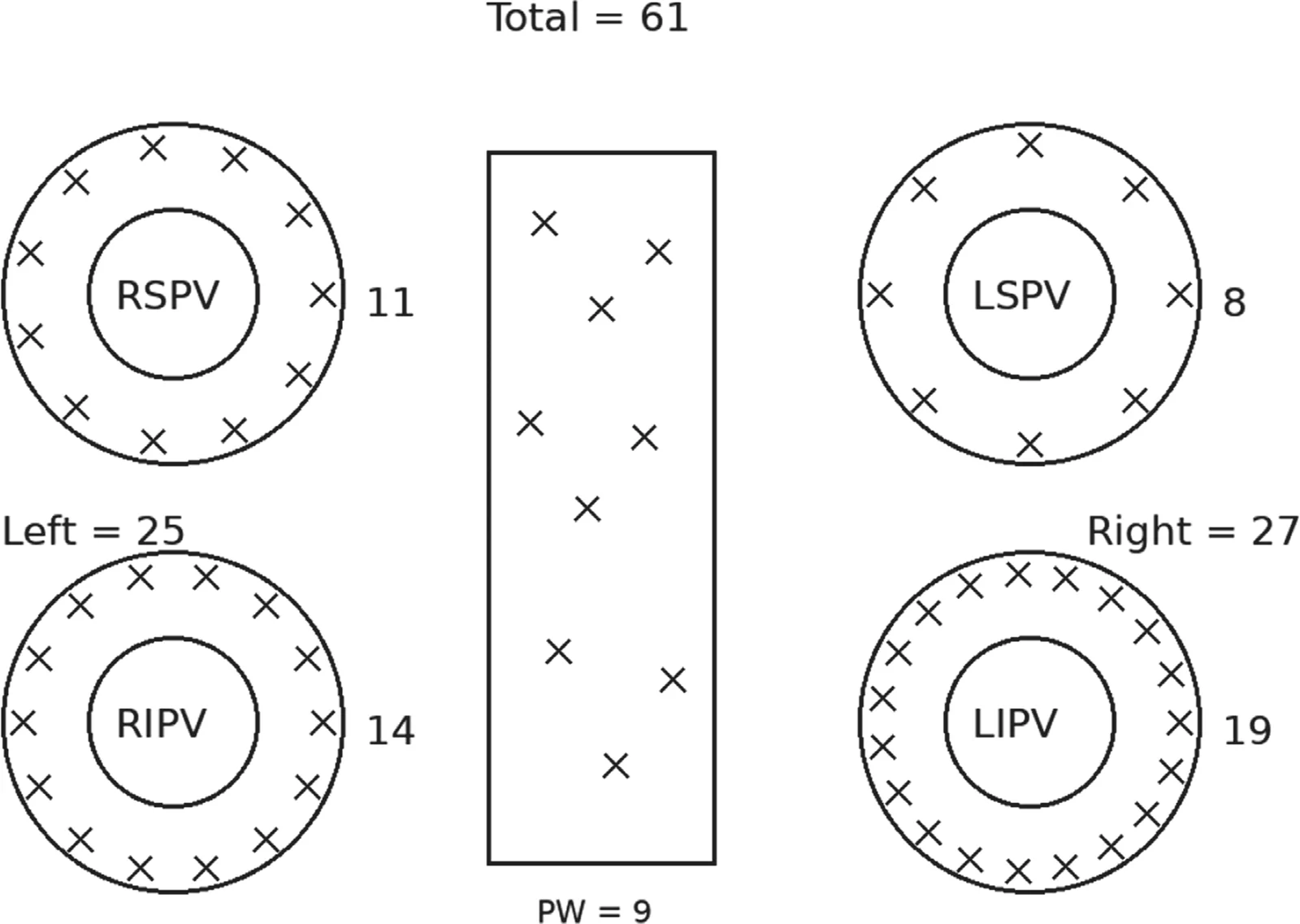
Anatomic distribution of pulsed field ablation lesions. Schematic representation of pulsed field ablation applications delivered circumferentially to the pulmonary veins and across the posterior left atrial wall. A total of 61 applications were delivered: right superior pulmonary vein (RSPV), 11; right inferior pulmonary vein (RIPV), 14; left superior pulmonary vein (LSPV), 8; left inferior pulmonary vein (LIPV), 19; and posterior wall (PW), 9. Crosses denote individual energy applications. *Abbreviations:* LIPV, left inferior pulmonary vein; LSPV, left superior pulmonary vein; PW, posterior wall; RIPV, right inferior pulmonary vein; RSPV, right superior pulmonary vein. Crosses denote individual energy applications.

The procedure was performed under general anesthesia. Using ultrasound-guided access, venous sheaths were inserted and a SoundStar™ intracardiac echocardiography catheter (J&J MedTech, New Brunswick, NJ, USA) was used for guidance, which showed no thrombus or effusion. A single transseptal puncture was performed using a Medtronic FlexCath sheath. Left atrial voltage mapping was completed using a Medtronic Octaray™ catheter on a CARTO™ system (J&J MedTech) **(Figure 2)**. PFA was delivered using a Medtronic PulseSelect™ catheter targeting all four pulmonary veins. Due to the presence of fractionated electrograms in the posterior wall, additional pulsed field applications were delivered across the superior and inferior pulmonary veins to achieve posterior wall isolation. A schematic of PFA lesion distribution is presented, with a total of 61 applications **(Figure 3)**. The entrance and exit blocks of all pulmonary veins and posterior wall were confirmed. No pericardial effusion was noted. The total fluoroscopy time was 3 min.

The patient tolerated the procedure well and was discharged the same day. She reported resolution of pre-procedure dizziness but noted new-onset mild shortness of breath. She stated that the shortness of breath had begun a few hours after the procedure and has persisted since. She described the shortness of breath as grade 2 on the Modified Medical Research Council (MMRC) scale. She also reported that it was worse with walking but denied any paroxysmal nocturnal dyspnea, edema, cough, or fever. Her labs and inflammatory markers were unremarkable. Chest radiographs performed a few days later demonstrated an elevated right hemidiaphragm, consistent with RPN palsy **(Figures 1A–1C)**. Conservative management with incentive spirometry was initiated. At the 12-day follow-up, her dyspnea had improved and was now described as grade 0 on the MMRC scale. Her ECG showed normal sinus rhythm with occasional premature atrial contractions. Flecainide was continued at a lower dose and dabigatran was maintained for anticoagulation. Though the patient’s symptoms improved, her 3-month chest radiograph showed persistent right hemidiaphragmatic palsy **(Figures 1D and 1E)**. At the 9-month follow-up, the patient was asymptomatic and therefore no further imaging was pursued. However, she will continue to follow up in the clinic for any symptoms.

## Discussion

Persistent phrenic nerve palsy following PFA has been previously reported.^4^ Although uncommon, this complication underscores that collateral neural injury may still occur despite the tissue-selective mechanism of electroporation. The present case adds procedural detail and follow-up imaging in the setting of PVI and posterior wall isolation using the PulseSelect™ system. PFA is designed to deliver electrical pulses that cause cell death through electroporation, ideally sparing non-cardiac tissues such as the phrenic nerve. Phrenic nerve paralysis is thought to be a very rare complication, as shown in the MANIFEST-PF registry, where, among 1568 patients who underwent ablation with the FARAPULSE™ PFA system, 0.4% developed transient phrenic nerve injury, recovering within a day, and one patient (0.06%) developed permanent phrenic nerve paralysis.^2^

During a recently published trial, the ElectroPulse study by Kamsani et al.,^6^ a novel PFA system with an 8-F, 10-electrode variable-loop steerable mapping and ablation catheter was employed during PVI and posterior wall isolation. The trial enrolled 30 patients, and no instances of phrenic nerve palsy were detected. Their study proposes that this new system can deliver higher voltages with lower rates of complications due to its integrated tissue contact monitoring system. However, they enrolled a notably a small number of patients, and larger trials are needed to truly assess whether this new catheter is less likely to cause phrenic nerve paralysis.^5^ The PulseSelect™ system, used in our case, was studied in the PULSED AF (“Pulsed Field Ablation to Irreversibly Electroporate Tissue and Treat AF”) pivotal trial of 300 patients, which demonstrated durable PVI without persistent phrenic nerve palsy.^3^ Similarly, the Varipulse™ system (J&J MedTech), fully integrated into the CARTO™ 3 platform, showed high acute success in early studies with no persistent phrenic involvement.^7^ Together, these data underscore that, although PFA is largely tissue-selective, vigilance for phrenic nerve injury remains important.

The mechanism of phrenic nerve injury is not completely understood. In animal studies assessing nerve damage from PFA, small hemorrhages and perineural edema were detected, though the nerve structure remained intact.^1,8^ Compound motor action potential (CMAP) monitoring has been proposed as a means to detect phrenic nerve dysfunction during PFA. However, no validated amplitude threshold exists to indicate when ablation should be ceased to prevent paralysis, and CMAP is not feasible when paralytic anesthetic agents are used.^9^ Ollitrault et al. conducted a study using the off-label pentaspline PFA catheter (Boston Scientific, Marlborough, MA, USA) for PVI and superior vena cava (SVC) isolation during atrial fibrillation catheter ablation. Notably, 67 of 105 patients (64%) had transient phrenic nerve stunning detected by CMAP during the procedure. However, none of these patients had phrenic nerve palsy post-procedure or later detected by chest radiography. The study proved transient phrenic nerve palsy particularly post-PFA application but demonstrated no permanent paralysis. The authors propose that the palsy is more electrophysiological as opposed to thermal injury.^10^ Further investigation is needed to better define the mechanism of phrenic nerve susceptibility during PFA.

The course of the RPN is anatomically variable and may place it at risk during right-sided ablation. After descending along the SVC, the RPN typically courses anterior to the right pulmonary veins; however, studies have demonstrated variability in its proximity to the right superior pulmonary vein (RSPV) antrum.^11,12^ In some individuals, the nerve lies immediately adjacent to the RSPV rather than remaining confined to the SVC territory, and phrenic capture from the RSPV region has been reported.^5,11,12^ This variability may explain cases of phrenic nerve injury during RSPV ablation even in the absence of SVC isolation.

Our case highlights that PVI with adjunct posterior wall isolation, even with PFA, is not immune to the complication of persistent phrenic nerve palsy. Fortunately, our patient’s symptoms improved with conservative measures.

## Conclusion

This case highlights that clinically significant phrenic nerve palsy with hemidiaphragmatic elevation may occur following PVI and posterior wall isolation using PFA. Vigilance with peri-procedural monitoring, awareness of anatomic proximity, and careful follow-up remain essential to optimize patient outcomes.

## References

[1] Maor E, Sugrue A, Witt C (2019). Pulsed electric fields for cardiac ablation and beyond: a state-of-the-art review. Heart Rhythm.

[2] Turagam MK, Neuzil P, Schmidt B (2023). Safety and effectiveness of pulsed field ablation to treat atrial fibrillation: one-year outcomes from the MANIFEST-PF registry. Circulation.

[3] Verma A, Haines DE, Boersma LV (2023). Pulsed field ablation for the treatment of atrial fibrillation: PULSED AF pivotal trial. Circulation.

[4] Oda Y, Nogami A, Igarashi D, Usui R, Uno K (2025). A case report of persistent phrenic nerve injury by pulsed field ablation: lessons from anatomical reconstruction and electrode positioning. HeartRhythm Case Rep.

[5] Howard B, Haines DE, Verma A (2022). Characterization of phrenic nerve response to pulsed field ablation. Circ Arrhythm Electrophysiol.

[6] Kamsani SH, Emami M, Young GD (2025). First-in-human experience of high-energy ElectroPulse pulsed field ablation: acute results for pulmonary veins and posterior wall isolation. Heart Rhythm.

[7] Mansour M, Maury P, Brugada J (2024). Pulsed field ablation with a variable-loop catheter: the inspIRE trial. Europace.

[8] Van Driel VJ, Neven K, Van Wessel H, Vink A, Doevendans PA, Wittkampf FH (2015). Low vulnerability of the right phrenic nerve to electroporation ablation. Heart Rhythm.

[9] Franceschi F, Koutbi L, Maille B, Deharo JC (2024). First electromyographic monitoring of a progressive phrenic nerve palsy in a pulsed field ablation procedure. HeartRhythm Case Rep.

[10] Ollitrault P, Chaumont C, Font J (2024). Superior vena cava isolation using a pentaspline pulsed-field ablation catheter: feasibility and safety in patients undergoing atrial fibrillation catheter ablation. Europace.

[11] Sánchez-Quintana D, Cabrera JA, Climent V, Farré J, Weiglein A, Ho SY (2005). How close are the phrenic nerves to cardiac structures? Implications for cardiac interventionalists. J Cardiovasc Electrophysiol.

[12] Sacher F, Monahan KH, Thomas SP (2006). Phrenic nerve injury after atrial fibrillation catheter ablation: characterization and outcome in a multicenter study. J Am Coll Cardiol.

